# Choline Attenuates Cardiac Fibrosis by Inhibiting p38MAPK Signaling Possibly by Acting on M_3_ Muscarinic Acetylcholine Receptor

**DOI:** 10.3389/fphar.2019.01386

**Published:** 2019-11-21

**Authors:** Lihui Zhao, Tingting Chen, Pengzhou Hang, Wen Li, Jing Guo, Yang Pan, Jingjing Du, Yuyang Zheng, Zhimin Du

**Affiliations:** ^1^Institute of Clinical Pharmacology, the Second Affiliated Hospital of Harbin Medical University (The University Key Laboratory of Drug Research, Heilongjiang Province), Harbin, China; ^2^Department of Clinical Pharmacology, College of Pharmacy, Harbin Medical University, Harbin, China; ^3^State Key Laboratory of Quality Research in Chinese Medicines, Macau University of Science and Technology, Macau, China

**Keywords:** M3 receptor, cardiac fibrosis, choline, collagen, p38MAPK

## Abstract

Choline has been reported to produce a variety of cellular functions including cardioprotection *via* activating M_3_ muscarinic acetylcholine receptor (M_3_R) under various insults. However, whether choline offers similar beneficial effects *via* the same mechanism in cardiac fibrosis remained unexplored. The present study aimed to investigate the effects of choline on cardiac fibrosis and the underlying signaling mechanisms, particularly the possible involvement of M_3_R. Transverse aortic constriction (TAC) mouse model was established to simulate the cardiac fibrosis. Transforming growth factor (TGF)-β1 treatment was employed to induce proliferation of cardiac fibroblasts *in vitro*. Choline chloride and M_3_R antagonist 4-diphenylacetoxy-*N*-methylpiperidine methiodide (4-DAMP) were used to unravel the potential role of M_3_R. Cardiac function was assessed by echocardiography and interstitial fibrosis was quantified by Masson staining. Protein levels of collagens I and III were determined by Western blot analysis. The role of M_3_R in the proliferation cardiac fibroblasts was validated by silencing M_3_R with specific small interference RNA (siRNA). Furthermore, the mitogen-activated protein kinase (MAPK) signaling pathway including p38MAPK and ERK1/2 as well as the TGF-β1/Smad pathway were analyzed. M_3_R protein was found abundantly in cardiac fibroblasts. M_3_R protein level, as identified by Western blotting, was higher in mice with excessive cardiac fibrosis and in TGF-β1-induced cardiac fibrosis as well. Choline significantly inhibited interstitial fibrosis, and this beneficial action was reversed by 4-DAMP. Production of collagens I and III was reduced after choline treatment but restored by 4-DAMP. Expression silence of endogenous M_3_R using siRNA increased the level of collagen I. Furthermore, the TGF-β1/Smad2/3 and the p38MAPK pathways were both suppressed by choline. In summary, choline produced an anti-fibrotic effect both *in vivo* and *in vitro* by regulating the TGF-β1/Smad2/3 and p38MAPK pathways. These findings unraveled a novel pharmacological property of choline linked to M_3_R, suggesting that choline regulates cardiac fibrosis and the associated heart diseases possibly by acting on M_3_R.

## Introduction

There are plenty of receptors that play an opposite or synergistic role in heart function (Pfleger et al., 2019). In the view of muscarinic acetylcholine receptors (MR), there are major subtypes (M_2_) and minor subtypes (M_1_, M_3_, maybe M_5_) in the heart, and the physiological and pathophysiological roles of these receptors have been uncovered (Colecraft et al., 1998; Shi et al., 1999; Hellgren et al., 2000; Brodde et al., 2001; Hardouin et al., 2002; Hang et al., 2013; Saternos et al., 2018). It has been reported that choline has some effects on M_3_ muscarinic acetylcholine receptor (M_3_R) in cardiac myocytes (Shi et al., 1999). This compound has been used as an agonist of muscarinic receptor in numerous published studies (Hang et al., 2009; Wang et al., 2009; Zhao et al., 2010; Liu L, 2017; Xu et al., 2019). Previous studies by our laboratory and others have demonstrated that activation of M_3_R protected against cardiac ischemia, cardiac hypertrophy, and arrhythmias (Pan et al., 2012; Liu et al., 2013). Specifically, activation of M_3_R by choline or overexpression of M_3_R in transgenic mice inhibits cardiac apoptosis, inflammation, calcium overload, and ion channel dysfunction (Yang et al., 2005; Liu et al., 2008; Liu et al., 2011; Wang et al., 2018). It is known that in the late phase of cardiac ischemia or hypertrophy, cardiac fibroblasts play an essential role in cardiac remodeling characterized by collagen overproduction and accumulation leading to cardiac interstitial fibrosis (Kong et al., 2014). While these studies primarily focused on the effects of M_3_R in cardiomyocytes, the function of M_3_R in cardiac fibroblasts and its potential role in cardiac fibrosis has not been exploited. Intriguingly, it has been documented that selective activation of M_3_R attenuates hepatic collagen deposition, bile ductule proliferation, and liver fibrosis (Khurana et al., 2013). In contrast, a study reported that cholinergic stimuli mediated by muscarinic receptors induced the proliferation of fibroblasts and myofibroblasts in airway (Pieper et al., 2007). A study reported by Organ et al. demonstrated that the inbred mice fed with choline has significantly enhanced cardiac fibrosis in a transverse aortic constriction (TAC) model (Organ et al., 2016). Another study conducted in a model of myocardial infarction reported that choline promotes cardiac fibrosis (Yang et al., 2019). All these studies suggest that M_3_R participates in the proliferation of fibroblasts and collagen production. However, the role of M_3_R in cardiac fibrosis remained controversial and inadequately addressed.

It has been well recognized that transforming growth factor (TGF)-β1/Smad cascade governs cardiac fibroblast proliferation and collagen secretion. For example, activation of the TGF-β1/Smad pathway promotes the growth of cardiac fibroblasts and collagen production (Zhang et al., 2016). In contrast, inhibition of TGF-β1/Smad limits the progression of cardiac fibrosis (Pan et al., 2011). In addition, many other signaling pathways have also been uncovered to participate in the development and progress of cardiac fibrosis. Among them, the mitogen-activated protein kinase (MAPK) pathway constituted by p38MAPK, ERK, and JNK is crucial to cardiac fibrosis and structural remodeling (Wang et al., 2016). Importantly, modulation of the MAPK pathway controls the pathological changes of cardiac fibrosis (Pan et al., 2011).

Taken together the above background information, we set up the present study focusing on the role of M_3_R in cardiac fibrosis and the underlying mechanisms. Our results demonstrated for the first time that choline significantly inhibits cardiac fibroblast proliferation and collagen secretion, and this anti-fibrotic property is likely ascribed to the inhibition of the TGF-β1/Smad and MAPK pathways.

## Materials and Methods

### Animals

Male Kunming mice and neonatal Sprague Dawley rats were purchased from the Animal Center of the Second Affiliated Hospital of Harbin Medical University (Harbin, China). The mice were maintained under standard animal room conditions (temperature, 21 ± 1°C; humidity, 55 to 60%), with food and water *ad libitum*. This study was conducted in strict accordance with the recommendations of the National Institutes of Health’s “Guidelines for the Care and Use of Laboratory Animals” (NIH publication, revised 2011). The protocol was approved by the Animal Care and Use Committee of Harbin Medical University.

A total of 32 mice of 20∼25 g were used in our study. The mice were anesthetized with 2,2,2-tribromoethanol (270 mg/kg) and TAC model with excessive cardiac fibrosis was established (Liu Y, 2017). The sham-operated control mice underwent the same surgical procedures without ligation of the aortic bundle. Three days after TAC, the mice were divided into four experimental groups (n = 8): sham, TAC, TAC + choline (14 mg/kg), TAC + choline + 4-DAMP (4-diphenylacetoxy-*N*-methyl-piperidine, 14 mg/kg choline, 0.7 µg/kg 4-DAMP). For co-administration of choline and 4-DAMP, 4-DAMP was injected 30 min before choline treatment and the administration method was intraperitoneal injection. After 8 weeks, cardiac function of the survived mice was examined by echocardiography. For molecular biology studies, the hearts were isolated and then quickly striped, cleared in cold buffer, and weighed after drying. The left ventricle preparations were stored frozen in a −80°C freezer for subsequent Western blot experiments.

### Cell Culture and Treatment

Hearts of neonatal SD rats (1–3 days) were cut into pieces and gathered in 50 ml centrifugal tube with 0.25% trypsin. The cell suspensions was collected in Dulbecco’s modified Eagle’s medium (DMEM, Corning, USA) supplemented with 10% fetal bovine serum, 100 U/ml penicillin, and 100 µg/ml streptomycin. It was then incubated in culture flasks for 2 h, to allow for fibroblasts to adhere to the bottom of the culture flasks. Unattached cardiomyocytes and other cells were removed. Isolated fibroblasts were incubated at 37°C in a humidified atmosphere of 5% CO_2_ and 95% air and nourished at an interval of every 2–3 days. The purity of cardiac fibroblasts used in our study was validated by staining specific marker vimentin using immunofluorescence (**Figure S1**). The cardiac fibroblasts were pre-treated with 3 nM 4-DAMP in the presence or absence of choline (1, 5, or 10 mM) for 1 h and then incubated with 20 ng/ml TGF-β1 for 48 h. 4-DAMP was dissolved in dimethyl sulfoxide (DMSO) and diluted to a final concentration of DMSO < 0.1%. The dose/concentration of M_3_ receptor 4-DAMP was selected according to previous studies (Wang et al., 2012; Zhao et al., 2013).

### Echocardiography and Histological Analysis

Mice were anesthetized mice before echocardiography. Both two-dimensional M-mode and three-dimensional Doppler echocardiography were performed by using the Vevo 770 imaging system (VisualSonics, Toronto, Canada) to evaluate cardiac diameter and the function of heart.

Echocardiographic parameters included left ventricular ejection fraction (LVEF), left ventricular shortening score (LVFS), the left ventricular end-diastolic diameter (LVIDd), and left ventricular end-systole diameter (LVIDs) For histological analysis, the hearts were fixed with 4% paraformaldehyde (pH 7.4) for 48 h. The tissue was soaked in paraffin, cut into 5-µm sections, and stained with Masson trichrome. Collagen deposition was quantified by Image-Pro Plus software (Media Cybernetics, Silver Spring, USA).

### Western Blot

Total protein samples were extracted from cardiac tissues and cardiac fibroblasts using lysis buffer. Protein sample (100 µg) was fractionated by sodium dodecyl sulfate polyacrylamide gel electrophoresis (10% polyacrylamide gels) and transferred to nitrocellulose membrane. The membrane was blocked with 5% nonfat milk at room temperature for 2 h. The membrane was then incubated with primary antibodies for collagen I (1:500), collagen III (1:500), M_3_R (1;500), TGF-β1 (1:500), total Smad2/3 (t-Smad2/3; 1:1,000), phosphorylated Smad1/3 (p-Smad2/3; 1:1,000), t-ERK (1:1,000), p-ERK (1:1,000), t-P38 (1:1,000), p-p38 (1:1,000), and glyceraldehyde 3-phosphate dehydrogenase (GAPDH) (1:1,000) on a shaking bed at 4°C overnight. The membrane was washed with PBS-Tween (0.5%) for three times and then incubated with secondary antibodies in the dark at room temperature for 1 h. Finally, the membranes were rinsed with PBS-T three times before being scanned by Imaging System (LI-COR Biosciences, Lincoln, NE, USA).

### Ribonucleic Acid Extraction and Real-Time Reverse Transcription Polymerase Chain Reaction

Total RNA (0.5 µg) was extracted from cardiac fibroblasts by using TRIzol^™^ Reagent (Thermo, USA) according to the manufacturer’s protocol. RNA concentration was measured and then reversely transcribed into complementary DNA. The messenger RNA (mRNA) levels of collagen I, collagen III, and TGF-β1 were determined using SYBR Green I incorporation method on LC480 Real-time PCR system (Roche, USA), with GAPDH as an internal control. The sequences of the primer pairs used in our study are as follow. Collagen I: forward (F): 5’-ATCAGCCCAAACCCCAAGGAGA-3’ and reverse (R): 5’-CGCAGGAAGGTCAGCTGGATAG-3,’ TGF-β1: F: 5’-CGCCTGCAGAGATTCAAGTCAAC-3’ and R: 5’-GTATCAGTGGGGGTCAGCAGCC-3,’ and GAPDH: F: 5’-TCCCTCAAGATTGTCAGCAA-3’ and R: 5’-AGATCCACAACGGATACATT-3.’

### Immunofluorescence

Cardiac fibroblasts were cultured in an incubator for 48 h, then washed with PBS. Next, the cells were fixed with 4% paraformaldehyde solution for 20 min, permeabilized with 1% Triton X-100 (prepared by PBS) at room temperature for 60 min, and incubated with goat serum (Solarbio, Beijing, China) at 37°C for 30 min, following three washes with PBS. Subsequently, the cells were incubated with collagen I antibody (1:500) at 4°C overnight, followed by incubation with fluorescence secondary antibody (1:500) and Alexa Fluor^®^ 488-conjugated goat anti-rabbit IgG (H + L) secondary antibody (Life Technologies) as a control in the dark at room temperature for 1 h. 4′,6-Diamidino-2-phenylindole (DAPI) (10 mg/ml, Beyotime, Haimen, China) was used for nuclear staining. Images were obtained using an Olympus microscope (Japan).

### Small Interference Ribonucleic Acid Transfection

Cardiac fibroblasts were transfected with an M_3_R small interference RNA (siRNA) or a scramble negative control (CTL) siRNA. Three siRNAs were used to screen the most potent sequence which was then used in subsequent experiments. The sequence of selected M_3_R siRNA was 5′-GCUACUGGCUGUGCUAUAUTTAUAUAGCACAGCCAGUAGCTT-3.′ Cells were transfected with 50 nM of siRNA using Lipofectamine 2000 (Invitrogen) for 6 h, before replacing with the medium containing 1% bovine serum. The cells were cultured for another 48 h. At 48 h after transfection, the cells were stimulated with TGF-β1 for 24 h and choline for an additional 24 h. The siRNA was constructed by GenePharma and transfected into cells according to the manufacturer’s instructions.

### Reagents

The recombinant human TGF-β1 was purchased from PeproTech (#100-21, NJ, USA). Choline chloride was purchased from Sigma (C7527, ≧98% purity, USA). 4-DAMP was purchased from Abcam (ab120144, USA). Anti-M_3_ receptor antibody was provided by Alomone (AMR-006; Israel). Antibodies against t-Smad2/3 (#8685), p-Smad2/3 (#8828), TGF-β1 (#3711), p38MAPK (#9212), p-p38MAPK (#9211), t-ERK1/2 (#4695), and p-ERK1/2 (#4370) were purchased from Cell Signaling Technology (CST, USA). Anti-collagen I antibody was purchased from Abcam (ab34710; Abcam, USA). Antibody against collagen III was purchased from Proteintech (13548-AP; Wuhan, China). Antibody against GAPDH was provided by ZSGB (TA-08; Beijing, China). Fluorescent secondary antibodies were purchased from LI-COR Biosciences (Lincoln, NE, USA).

### Statistical Analysis

Data are presented as mean ± SEM. Comparison between two groups was performed using an unpaired Student’s t-test. Comparisons among multiple groups were determined by one-way ANOVA followed by a *post hoc* Tukey test. The randomized block ANOVA (repeated measures ANOVA) was used for western blot data with a control value of 1 and no SEM as described previously (Lew, 2007). A value of p < 0.05 was considered statistically significant.

## Results

### Protein Level of M_3_ Muscarinic Acetylcholine Receptor in Transforming Growth Factor Beta 1-Induced Cardiac Fibroblasts

**Figure 1:**
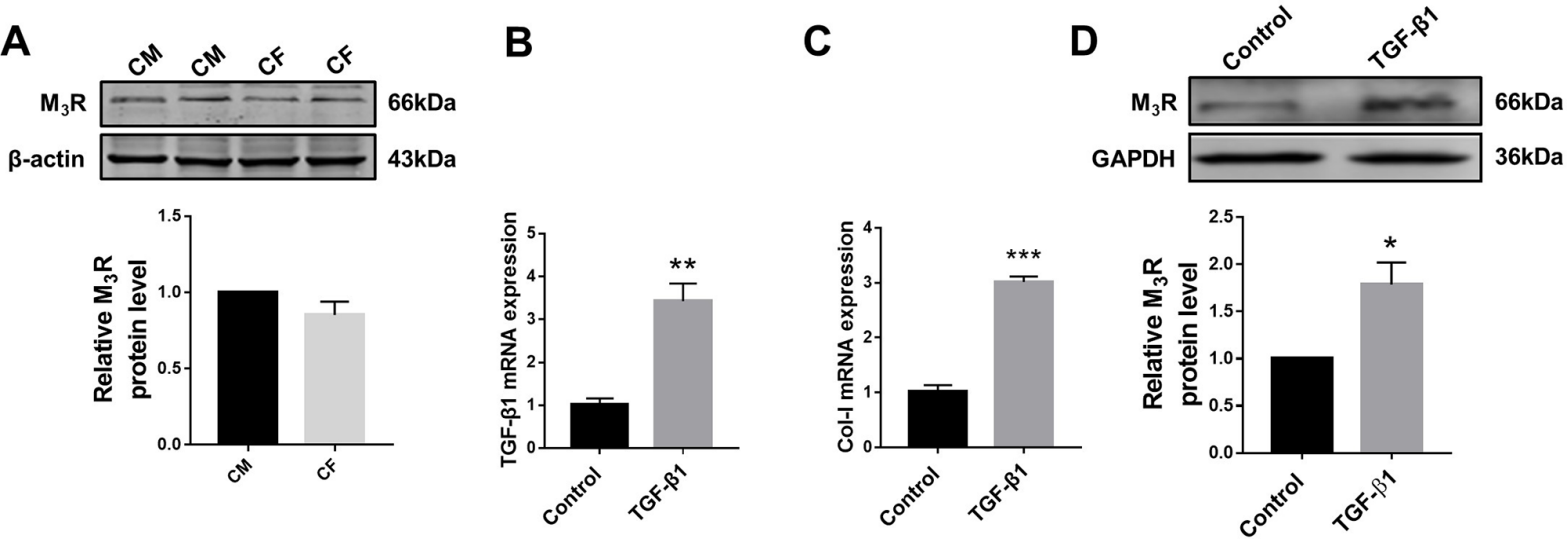
Protein level of M3 muscarinic acetylcholine receptor (M3R) in transforming growth factor beta 1 (TGF-β1)-induced cardiac fibroblasts (CF). **(A)** Protein level of M3R in CF and cardiomyocytes (CM). n = 3. **(B)** Effect of TGF-β1 (20 ng/ml, 48 h) on messenger RNA level of TGF-β1. **p < 0.01 *vs.* Ctrl, n = 3. **(C)** Effect of TGF-β1 (20 ng/ml, 48 h) on mRNA level of collagen I. ***p < 0.001 *vs.* Ctrl, n = 3. **(D)** Effect of TGF-β1 (20 ng/ml, 48 h) on protein level of M_3_R in CF. *p < 0.05 *vs.* Ctrl, n = 4.

Previous studies demonstrated that M_3_R is expressed in cardiomyocytes; however, whether this subtype of MR is also expressed in cardiac fibroblasts remained unknown. We therefore firstly detected protein level of M_3_R in cardiac fibroblasts using cardiomyocytes as a positive control group. As shown in **Figure 1A**, protein level of M_3_R in cardiac fibroblasts was within the same range as that in cardiomyocytes. We then treated cardiac fibroblasts with 20 ng/ml TGF-β1 for 48 h to promote their proliferation. The mRNA levels of TGF-β1 and collagen I were significantly higher in TGF-β1-treated cells than in non-treated control cells (**Figures 1B, C**). Moreover, TGF-β1 markedly elevated the protein level of M_3_R (**Figure 1D**). These findings support that M_3_R is expressed in cardiac fibroblasts and can be activated in response to TGF-β1 stimulation.

### Effects of Choline on Protein Levels of Collagen in Cardiac Fibroblasts

**Figure 2:**
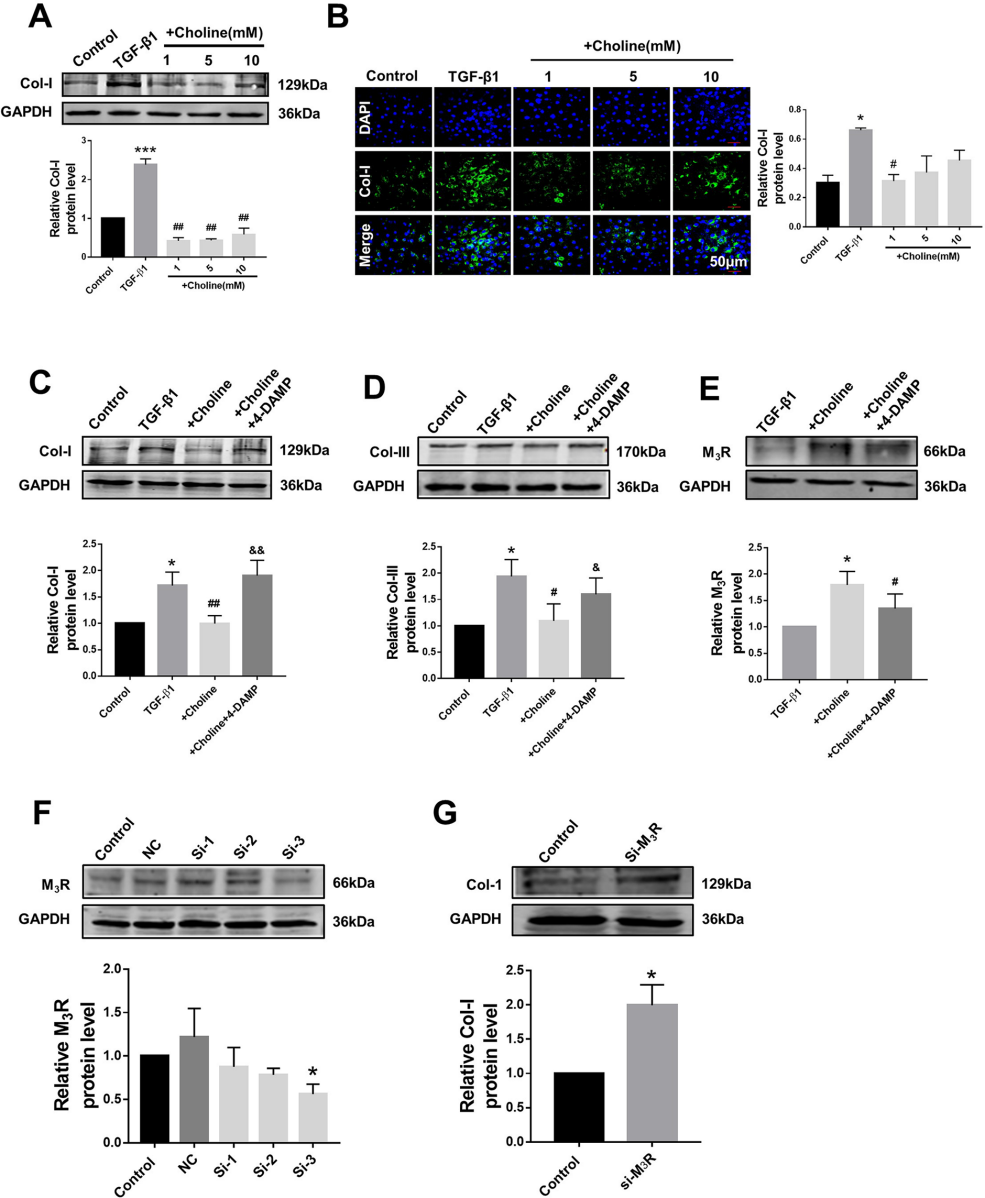
Effects of choline and 4-diphenylacetoxy-*N*-methylpiperidine methiodide (4-DAMP) on transforming growth factor beta 1 (TGF-β1)-induced collagen secretion. **(A)** Effects of different concentrations of choline (1, 5, 10 mM) on protein level of collagen I. ***p < 0.001 *vs.* Ctrl, ^##^ *p* < 0.01 *vs.* TGF-β1, n = 5. **(B)** Immunofluorescence for collagen I in cardiac fibroblasts (CF). Images were obtained using fluorescence microscopy. Blue fluorescence indicates 4′,6-diamidino-2-phenylindole, green fluorescence indicates collagen I, scale bars: 50 μm. *p < 0.05 *vs.* Ctrl, ^#^ *p* < 0.05 *vs.* TGF-β1, n = 3. **(C)** Effect of choline on collagen I protein level in the different experimental groups. *p < 0.05 *vs.* Ctrl, ^##^ *p* < 0.01 *vs.* TGF-β1, ^&&^ *p* < 0.01 *vs.* choline, n = 7. **(D)** Effect of choline on collagen III protein level in the different experimental groups. *p < 0.05 *vs.* Ctrl, ^#^ *p* < 0.05 *vs.* TGF-β1, ^&^ *p* < 0.05 *vs.* choline, n = 6. **(E)** Effects of choline (1 mM) and 4-DAMP (3 nM) on protein level of M_3_ muscarinic acetylcholine receptor (M_3_R) in TGF-β1-treated CF. *p < 0.05 *vs.* Ctrl, ^#^ *p* < 0.05 *vs.* TGF-β1, n = 5. **(F)** Protein level of M_3_R after transfecting with three fragments of M_3_R-siRNA. *p < 0.05 *vs.* Ctrl, n = 4. **(G)** Protein level of collagen I after transfecting M_3_R-siRNA-3. *p < 0.05 *vs.* Ctrl, n = 5.

In order to investigate the effects of M_3_R on the proliferation of cardiac fibroblasts, we measured the protein levels of collagen I after treatment with a muscarinic acetylcholine receptor agonist choline at concentrations of 1, 5, and 10 mM. Both western blot and immunofluorescence results showed that collagen I was significantly decreased by 1 mM choline (**Figures 2A, B**). And higher concentration of choline (5 and 10 mM) did not impose further inhibitory effects on collagen I levels. We therefore used 1 mM choline for subsequent experiments. As shown in **Figures 2C, D**, compared with the TGF-β1 group, collagens I and III were significantly decreased by 1 mM choline, and this effect was abolished by adding 4-DAMP, a specific antagonist of M_3_R. These data suggested that activation of M_3_R significantly inhibits the secretion of collagen in cardiac fibroblasts.

Furthermore, the protein level of M_3_R was further up-regulated by choline in cardiac fibroblasts pretreated with TGF-β1, which was partially but significantly reversed by 4-DAMP (**Figure 2E**). These results suggest that M_3_R up-regulation and M_3_R activation are both involved in inhibiting collagen production in TGF-β1-treated cardiac fibroblasts.

Because choline and 4-DAMP are not highly specific ligands for M_3_R, siRNA of M_3_R siRNA was used by transfection to specifically silence the expression of M_3_R, and to validate the effects of M_3_R on the proliferation of cardiac fibroblasts. We examined three M_3_R siRNAs and selected the one with the highest silencing efficacy from them for subsequent experiments (**Figure 2F**). As expected, silence of M_3_R promoted collagen production in cardiac fibroblasts treated with M_3_R siRNA compared with the control group (**Figure 2G**).

### Effects of Choline on Transverse Aortic Constriction-Induced Cardiac Dysfunction in Mice

**Figure 3:**
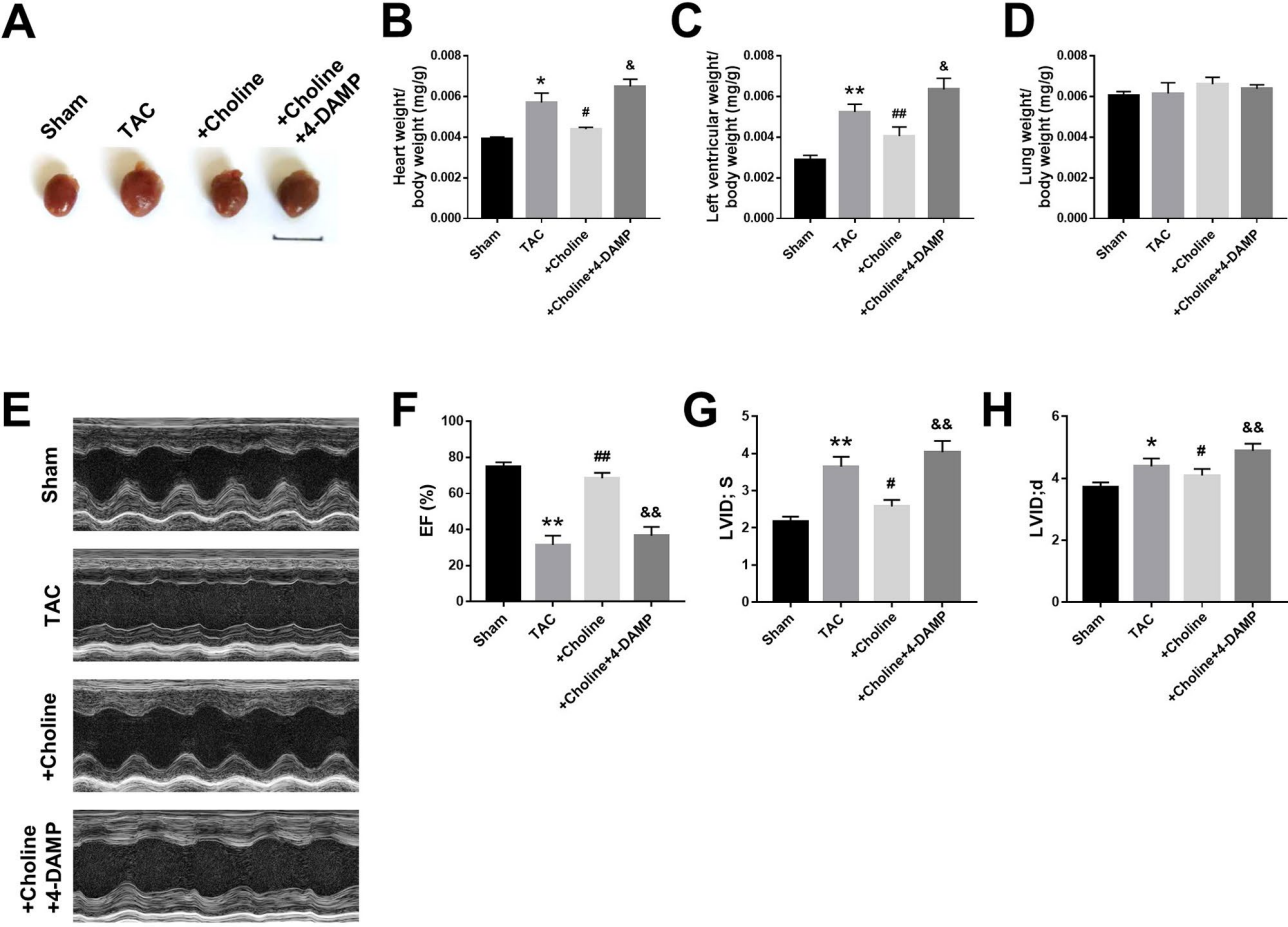
Effects of choline on cardiac function of transverse aortic constriction (TAC) mice model. **(A)** Representative hearts from sham, TAC, and the mice induced by TAC treated with choline (14 mg/kg per day) and 4-DAMP (0.7 μg/kg/day) for 8 weeks. **(B)** The ratio of heart weight/body weight (BW). **(C)** The ratio of left ventricular weight (LVW)/BW. **(D)** The ratio of lung weight/body weight. **(E)** Representative echocardiographic images of mouse hearts in each group. **(F)** Left ventricular ejection fraction (LVEF). **(G)** Systolic left ventricular internal diameter (LVID, s). **(H)** Diastolic left ventricular internal diameter (LVID, d). *p < 0.05, **p< 0.01 *vs.* sham, ^#^ *p* < 0.05, ^##^ *p* < 0.01 *vs.* TAC, ^&^ *p* < 0.05, ^&&^ *p* < 0.01 *vs.* choline, n = 6.

As shown in **Figure 3A**, the heart size of TAC mice was obviously larger than sham control mice but was markedly reduced by choline. The effect of choline was abrogated by 4-DAMP. Consistently, both the ratios of heart weight to body weight and left ventricular weight to body weight of TAC mice were decreased by choline, which was reversed by 4-DMAP (**Figures 3B, C**). No significant difference of the ratio of lung weight/body weight was found (**Figure 3D**). Echocardiographic data revealed that LVEF was increased, while the thickness of the posterior wall of the left ventricle was significantly reduced by choline. These effects were weakened by 4-DAMP pretreatment (**Figures 3F–H**), suggesting that M_3_R antagonism accounts at least partially for the cardiac dysfunction in TAC mice and choline improves the impaired cardiac function.

### Effects of Choline on Cardiac Interstitial Fibrosis

**Figure 4:**
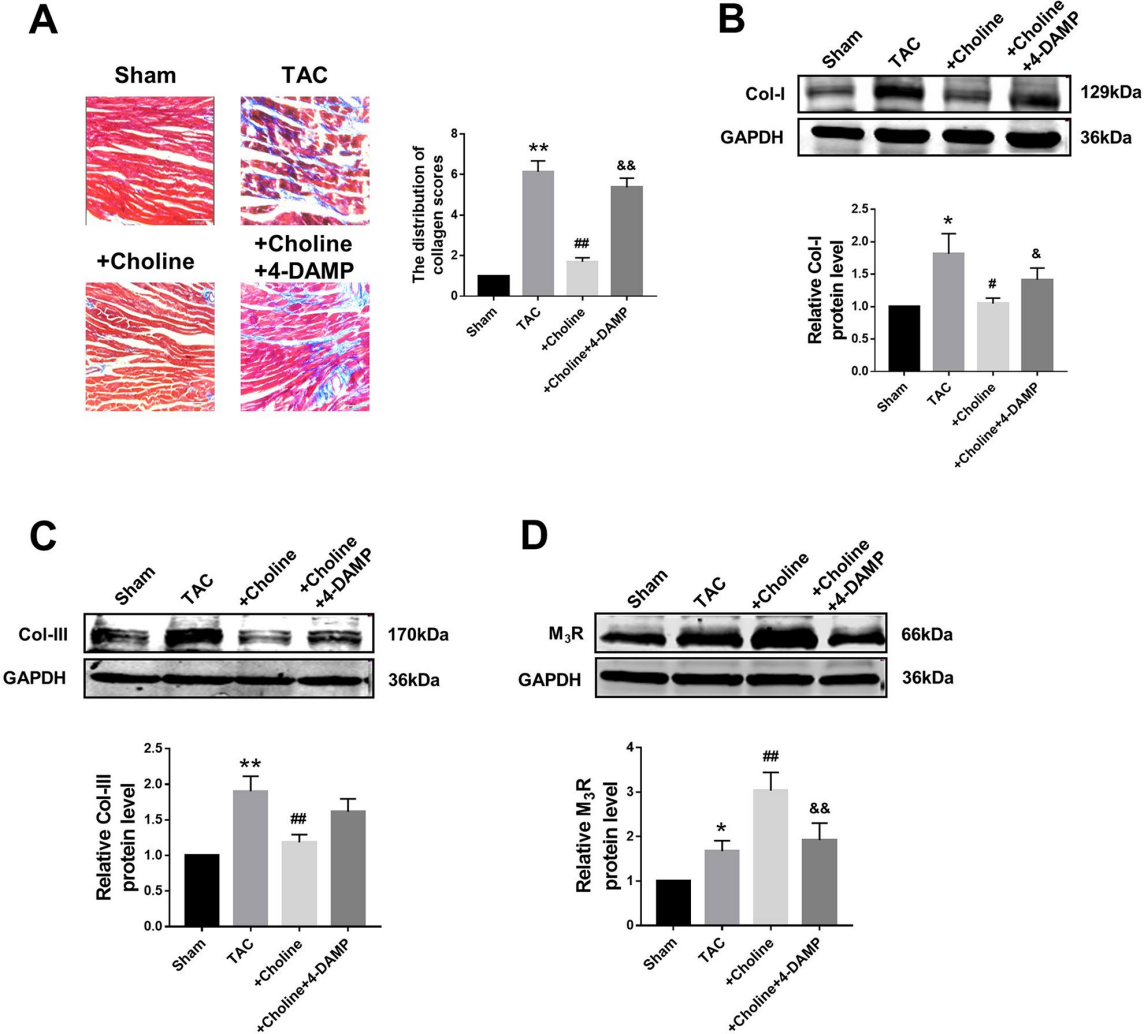
Effects of choline on transverse aortic constriction (TAC)-induced myocardial fibrosis. **(A)** Representative heart section with Masson staining in the experimental groups, scale bar: 100 μm. **p < 0.01 *vs.* sham, ^##^ *p* < 0.01 *vs.* TAC, ^&&^ *p* < 0.01 *vs.* choline, n = 5. **(B)** Effects of choline and 4-diphenylacetoxy-*N*-methylpiperidine methiodide (4-DAMP) on the protein level of collagen I. *p < 0.05 *vs.* sham, ^#^ *p* < 0.05 *vs.* TAC, ^&^ *p* < 0.05 *vs.* choline, n = 8. **(C)** Effects of choline and 4-DAMP on the protein level of collagen III. **p < 0.01 *vs.* sham, ^##^ *p* < 0.01 *vs.* TAC, n = 8. **(D)** Cardiac M_3_ muscarinic acetylcholine receptor protein level after TAC surgery and choline or 4-DAMP treatment. *p < 0.05 *vs.* sham, ^##^ *p* < 0.01 *vs.*TAC, ^&&^ *p* < 0.01 *vs.* choline, n = 8.

Masson staining shown in **Figure 4A** revealed that choline treatment decreased the collagen-enriched area and attenuated the inflammatory cell infiltration of myocardial fibrosis induced by TAC, which was reversed by 4-DAMP. Meanwhile, the protein levels of collagens I and collagen III were found significantly higher in the TAC group than in the sham group, and this TAC-induced collagen deposition was essentially inhibited in the choline group (**Figures 4B, C**). Meanwhile, protein level of M_3_R was increased in TAC mice compared to that in sham mice, and this upregulation was further exaggerated by choline but repressed by 4-DAMP (**Figure 4D**). These results suggest that choline suppresses, whereas M_3_R inhibition facilitates cardiac fibrosis.

### Suppressive Effects of Choline on the Transforming Growth Factor Beta 1/Smad Pathway in Cardiac Fibroblasts and Transverse Aortic Constriction Mice

**Figure 5:**
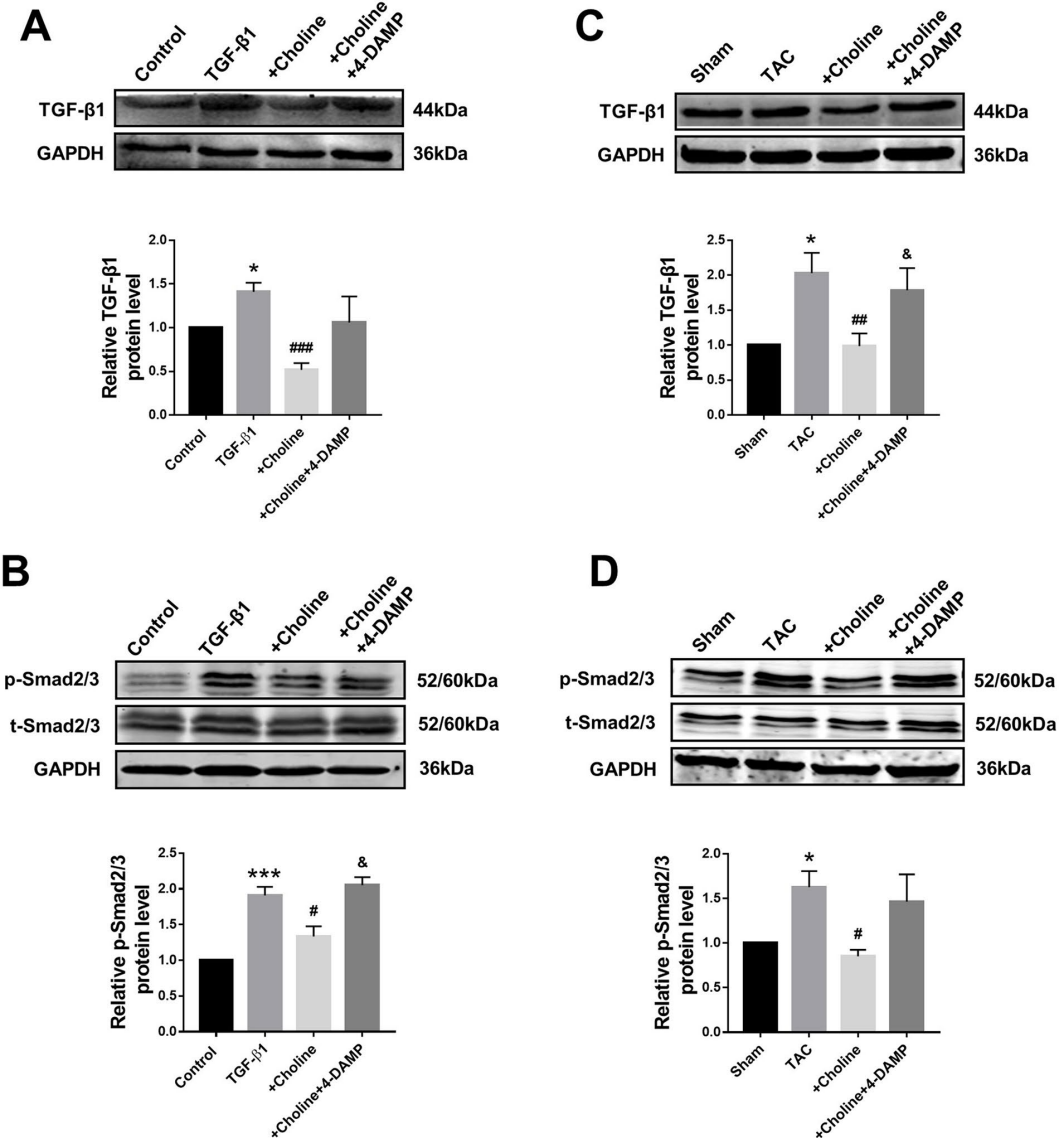
Effects of choline and 4-diphenylacetoxy-*N*-methylpiperidine methiodide (4-DAMP) on protein levels of transforming growth factor beta 1 (TGF-β1)/Smad2/3. **(A)** Effect of choline and 4-DAMP on TGF-β1 protein level in TGF-β1-induced cardiac fibroblasts (CF). *p < 0.01 *vs.* Ctrl, ^###^ *p* < 0.001 *vs.* TGF-β1, n = 5. **(B)** Effect of choline and 4-DAMP on p-Smad2/3 protein level in TGF-β1-induced CF. ***p < 0.001 *vs.* Ctrl, ^#^ *p* < 0.05 *vs.* TGF-β1, ^&^ *p* < 0.05 *vs.* choline, n = 6. **(C)** Effects of choline and 4-DAMP on the protein level of TGF-β1 in transverse aortic constriction (TAC) mice hearts. *p < 0.05 *vs.* sham, ^##^ *p* < 0.01 *vs.* TAC, ^&^ *p* < 0.05 *vs.* choline, n = 6. **(D)** Effects of choline and 4-DAMP on the protein level of p-smad2/3 in TAC mice hearts. *p < 0.5 *vs.* sham, ^#^ *p* < 0.05 *vs.* TAC, n = 4.

The classical TGF-β1/Smad signaling pathway is a key determinant of cardiac fibrogenesis. Our Western blot results showed that the protein levels of TGF-β1 and Smad2/3 were significantly lower in the choline group than in the TGF-β1 group, 4-DAMP abolished the effects of choline (**Figures 5A, B**). Similar results were consistently observed in TAC mice: the protein levels of TGF-β1 and p-Smad2/3 were substantially increased in TAC mice relative to those in sham control group. Moreover, choline mitigated the TAC-induced upregulation TGF-β1 and p-Smad2/3 levels and addition of 4-DAMP nearly entirely abolished the effects of choline (**Figures 5C, D**).

### Suppressive Effects of Choline on Mitogen-Activated Protein Kinase Signaling in Cardiac Fibroblasts and Transverse Aortic Constriction Mice

**Figure 6:**
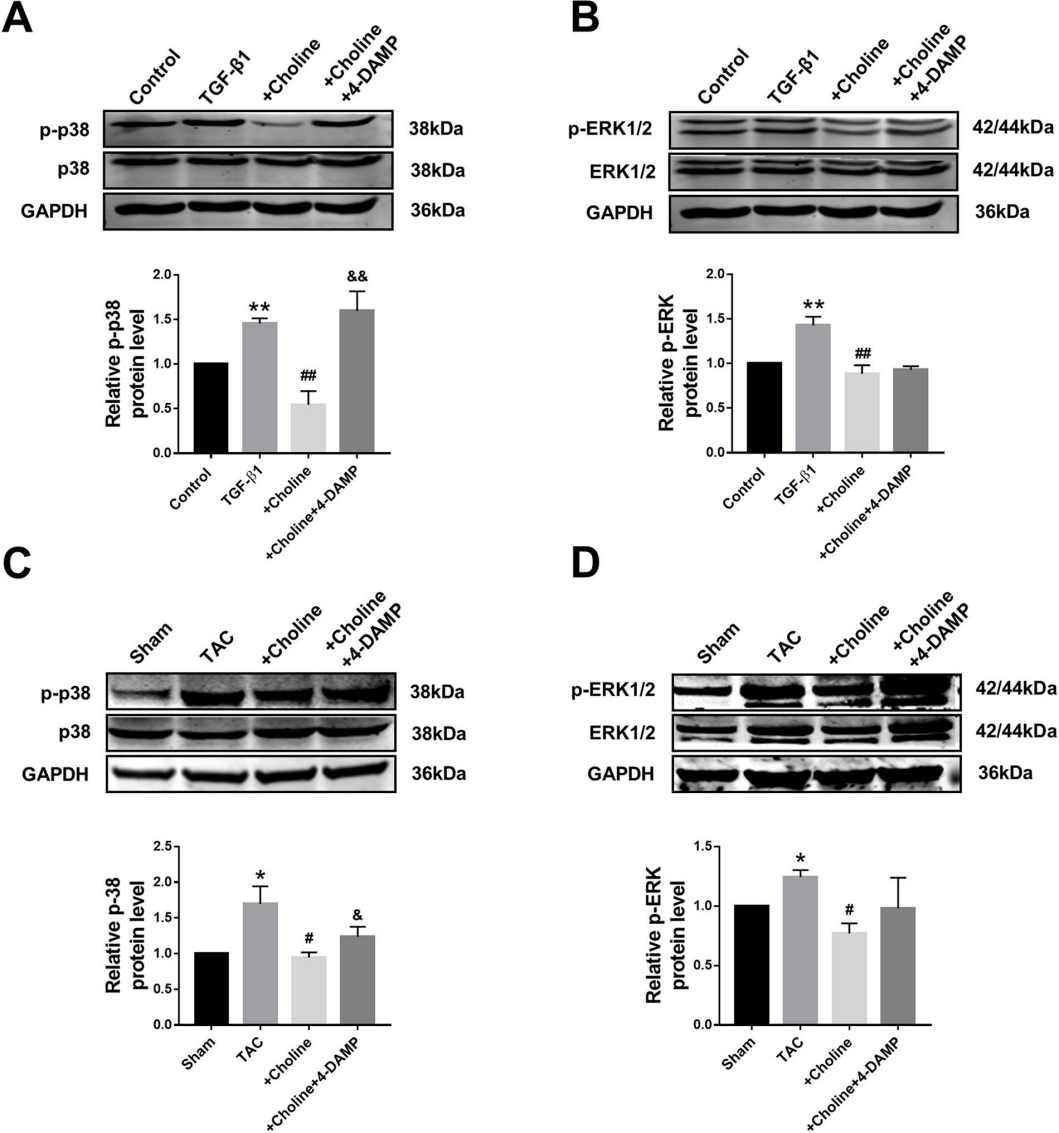
Effect of choline and 4-diphenylacetoxy-*N*-methylpiperidine methiodide (4-DAMP) on protein levels of p38MAPK and ERK1/2. **(A)** Effect of choline and 4-DAMP on p-p38 protein level in transforming growth factor beta 1 (TGF-β1)-induced cardiac fibroblasts (CF). **p < 0.01 *vs.* Ctrl, ^##^ *p* < 0.01 *vs.* TGF-β1, ^&&^ *p* < 0.01 *vs.* choline, n = 5. **(B)** Effect of choline and 4-DAMP on p-ERK1/2 protein level in TGF-β1-induced CF. **p < 0.01 *vs.* Ctrl, ^##^ *p* < 0.01 *vs.* TGF-β1, n = 6. **(C)** Effects of choline and 4-DAMP on the protein level of p-p38 in transverse aortic constriction (TAC) mice hearts. *p < 0.05 *vs.* sham, ^#^ *p* < 0.05 *vs.* TAC, ^&^ *p* < 0.05 *vs.* choline, n = 5. **(D)** Effects of choline and 4-DAMP on the protein level of p-ERK in TAC mice hearts. *p < 0.05 *vs.* sham, ^#^ *P*< 0.05 *vs.* TAC, n = 4.

It is well known that the MAPK signaling pathway plays an important role in myocardial ischemia and cardiac hypertrophy. We therefore next explored the potential relationship between M_3_R and MAPK signaling. On one hand, the ratio of p-p38/t-p38, and of p-ERK1/2/t-ERK1/2 in the choline group were significantly lower than in the TGF-β1 group, while 4-DAMP eliminated the suppressive effect of choline on p-p38, it failed to affect the effect of choline on p-ERK1/2 (**Figures 6A, B**). On the other hand, protein levels of p-p38MAPK and p-ERK were increased in the TAC group compared with the sham group, and choline abrogated such increases while 4-DAMP partially prevented the suppressive effect of choline (**Figures 6C, D**). These findings suggesting that M_3_R activation inhibits activation of p38 and ERK1/2 thereby MAPK signaling.

## Discussion

Although accumulating evidence has supported that M_3_R is expressed in cardiomyocytes of both human and rodents (Gadbut and Galper, 1994; Hellgren et al., 2000; Wang et al., 2001; Stengel et al., 2002; Willmy-Matthes et al., 2003; Wang et al., 2004; Abramochkin et al., 2012), its expression, and function in cardiac fibroblasts remained vaguely understood. To shed light on this issue, we conducted the present study focusing on the possible role M_3_R in regulating proliferation and collagen production of rat cardiac fibroblasts *in vitro* and cardiac fibrosis in TAC mice *in vivo*. The results demonstrated for the first time that M_3_R is expressed in cardiac fibroblasts of rodents, and either pharmacological inhibition or expression silence of M_3_R favors, while choline that has the potential to activate M_3_R limits cardiac fibrosis by inhibiting p38MAPK signaling.

It has been accepted that M_2_R is not the only functional subtype muscarinic and nicotinic acetylcholine receptors (mAChRs) in the heart (Saternos et al., 2018). Numerous studies have reported that M_3_R plays an important role in heart diseases (Filatova et al., 2017; Xue et al., 2017). However, these studies primarily focused on cardiomyocytes and no studies have reported the expression and function of M_3_R in cardiac fibroblasts, though it has been shown that M_3_R is expressed in certain types of non-cardiac fibroblasts. For example, Pieper et al. demonstrated that M_1_, M_2_, and M_3_ receptors are expressed at the mRNA level in lung fibroblasts. They also found that cholinergic stimuli mediated by muscarinic receptors cause remodeling in chronic airway disease (Pieper et al., 2007). Reina et al. reported that pilocarpine activates muscarinic M_1_ and M_3_ receptors, which promotes apoptosis in human skin fibroblast cells (Reina et al., 2010). Here, we demonstrate that M_3_R proteins are presented in both cardiac fibroblasts and cardiomyocytes with similar abundance. Previous studies by ours and other laboratories suggest that choline produces a protective effect against cardiac hypertrophy by activating M_3_R (Wang et al., 2012; Liu et al., 2013; Xu et al., 2019). For example, Xu et al. observed significant attenuation of cardiac fibrosis after choline treatment in cardiac hypertrophy model (Xu et al., 2019). However, the mechanism for the anti-fibrotic effect of choline is unclear. The present study provided direct evidence of the anti-fibrotic effect of choline *via* acting on M_3_R. This note was well supported by the data we obtained using M_3_R-selective antagonist 4-DAMP and M_3_R-specific siRNA. Notably, we found that 1 mM choline produced maximum anti-fibrotic effects and increasing concentrations up to 10 mM did not yield further effects. As already mentioned earlier, two published studies demonstrated that choline promotes cardiac fibrosis in mouse models of TAC and myocardial infarction as well (Organ et al., 2016; Yang et al., 2019), which is in contradiction to the findings presented in the present study. The discrepancy could be explained by the following possibilities. First, in the two published studies, the authors ascribed the results to the microbiome conversion of choline to trimethylamine N-oxide (TMAO) as the animals were fed with choline diet; in other words, the observed enhancement of cardiac fibrosis by choline diet is primarily caused by TMAO. However, such an explanation might not be applied to our case because in our *in vivo* experiments, choline chloride was intraperitoneally injected into mice, and it is unlikely that choline undergoes microbiome conversion to TMAO. Second, in our *in vitro* study, fibroblasts were incubated directly with choline and again it is unlikely for choline to convert to TMAO either. Third, the fact that the beneficial action of choline was efficiently reversed by 4-DAMP suggests that in our models, choline likely acts directly on M_3_R without an involvement of TMAO or other factors.

It is well established that the MAPK pathway plays an important role in cardiac fibrosis by modulating the proliferation and differentiation of cardiac fibroblasts (Chang et al., 2018). Cardiac fibroblast-specific p38α MAPK causes cardiac ventricular remodeling and fibrosis promotes cardiac hypertrophy *via* regulating interleukin-6 signaling. Conversely, fibroblast-specific p38α knockout mice exhibits marked protection against myocardial injury and fibrosis (Bageghni et al., 2018). Moreover, a previous study also suggests that activation of M_3_R by choline relieves cardiac ischemia and hypertrophy by inhibiting p38MAPK signaling (Wang et al., 2012). The present study shows that the negative impact of M_3_R on p38MAPK also exists in cardiac fibroblasts.

In the present study, we used choline as an agonist of M_3_R; however, it must be noted that though the ability of choline to activate M_3_R has been documented by numerous studies, this compound is not a selective M_3_R agonist. Instead, choline has been shown to produce a variety of cellular functions. For instance, it was demonstrated that choline can be uptaken by transporters and then it activates sigma-1 receptors (Sig-1R), a group of integral membrane proteins of endoplasmic reticulum and potentiates Ca^2+^ signals (Brailoiu et al., 2019). Evidence was provided in this study that choline mimics other Sig-1R agonists by potentiating Ca^2+^ signals evoked by the inositol 1,4,5-trisphosphate receptors. The authors conclude that choline is an endogenous agonist of Sig-1Rs linking extracellular stimuli to Ca^2+^ signals. It is also noted that this study reports a choline displacement of Sig-1R specific radioligand binding by [^3^H](+)-pentazocine with pKi value of around 3.3 mM, which is essentially in the same range of choline for M_3_R as reported by Shi et al. (1999). Together these findings, it appears that choline is a non-selective agonist for both M_3_R and Sig-1R and maybe for other receptors too. Another study demonstrated that Sig-1R knockout mice have significantly increased cardiac fibrosis and collagen deposition in the hearts, indicating an involvement of Sig-1R in regulating cardiac fibrosis (Abdullah et al., 2018). A most recent report demonstrates that BD1047 (an antagonist of Sig-1R can cause an increase in atrial fibrosis contributing to exacerbating atrial fibrillation (Ye et al., 2019). Due to the present lack of subtype-selective mAChR agonists, we employed choline as a partial agonist of M_3_R in the present study. Precaution must therefore be taken in interpreting our results obtained with choline in terms of the mechanism of action; in other words, the present study does not exclude the possibility of choline to interact with Sig-1R and produce the anti-fibrotic action. Nonetheless, it should also be noted that there has not been any evidence for the presence of Sig-1R in cardiac fibroblasts, with which the present study was conducted.

The study reported by Jaiswal et al. in 1989 (Jaiswal et al., 1989) stands the first evidence for functional M_3_R in mammalian hearts, which was verified the same group in 1996 in ventricular myocytes of rabbit hearts (Kan et al., 1996). The existence of M_3_R in cardiomyocytes has been recognized by several more confirmative studies from multiple research groups with pharmacological, functional, and molecular evidence (Hellgren et al., 2000; Oberhauser et al., 2001; Pönicke et al., 2003; Wang et al., 2004). Nevertheless, whether M_3_R also exists in cardiac fibroblasts remained unknown prior to the present study; thus, we present here the first evidence for the expression and function of M_3_R in cardiac fibroblasts. Though our study does not provide conclusive evidence, the most rational and objective explanation of our data is the participation of M_3_R in cardiac fibrosis.

In addition to M_2_R and M_3_R, the heart also expressed other subtypes of mAChRs, including M_1_R (Colecraft et al., 1998; Hardouin et al., 2002) and M_4_R (Colecraft et al., 1998; Shi et al., 2004; Wang et al., 2004). The presence of M_1_R and M_2_R proteins on the surface membrane of the cultured rat ventricular myocytes was confirmed by immunofluorescence (Colecraft et al., 1998). The study suggests that the positive chronotropic effect of mAChR activation on the contractions is mediated through the M_1_R coupled through Gq to phospholipase C-induced phosphoinositide hydrolysis. In contrast, a study suggests the absence of M_1_R expression in mouse heart (Hardouin et al., 2002). This conclusion was primarily based on the following two pieces of evidence. First, basal values of heart rate, developed left ventricular pressure, left ventricular dP/dt_max_, and mean blood pressure are similar between wild type and M_1_R-knockout mice. Second, administration of M_1_R-selective agonist McN-A-343 increases hemodynamic function in wild-type mice but fails to cause any changes in M_1_R knockout mice.

Regarding the statistical analysis applied to western blots, calculating the average of all data from different batches of experiments represents an appropriate and more powerful analysis. However, in our study, western blot experiments presented too much variability in batches of experiments, so we normalized the data to control in each western blot gel firstly, then analyzed the normalized data from different batches. This might represent a limitation of the study, which should be validated in future study.

In conclusion, our study suggests that choline significantly inhibits cardiac fibroblast proliferation and collagen secretion likely *via* activating M_3_R with the functional role of which being associated with the TGF-β1/Smad and p38MAPK pathways.

## Data Availability Statement

The raw data supporting the conclusions of this manuscript will be made available by the authors, without undue reservation, to any qualified researcher.

## Ethics Statement

The animal study was reviewed and approved by Ethical Committee of Harbin Medical University.

## Author Contributions

LZ, TC: acquisition, analysis, and interpretation of data. PH: analysis and interpretation of data, and manuscript writing. JG, WL, YP, JD, YZ: acquisition of data. ZD: conception and design, manuscript revision, and final approval of manuscript.

## Funding

This work was supported by National Natural Science Foundation of China (No. 81673424 and 81300080).

## Conflict of Interest

The authors declare that the research was conducted in the absence of any commercial or financial relationships that could be construed as a potential conflict of interest.
