# Supplementary material for: Choline Attenuates Cardiac Fibrosis by Inhibiting p38MAPK Signaling Possibly by Acting on M3 Muscarinic Acetylcholine Receptor
Source: Front Pharmacol. 2019 Nov 21;10:1386. doi: 10.3389/fphar.2019.01386 (PMC6900736; doi:10.3389/fphar.2019.01386)
Supplement: Supplementary file 1 [file Image_1.pdf]

Supplemental data

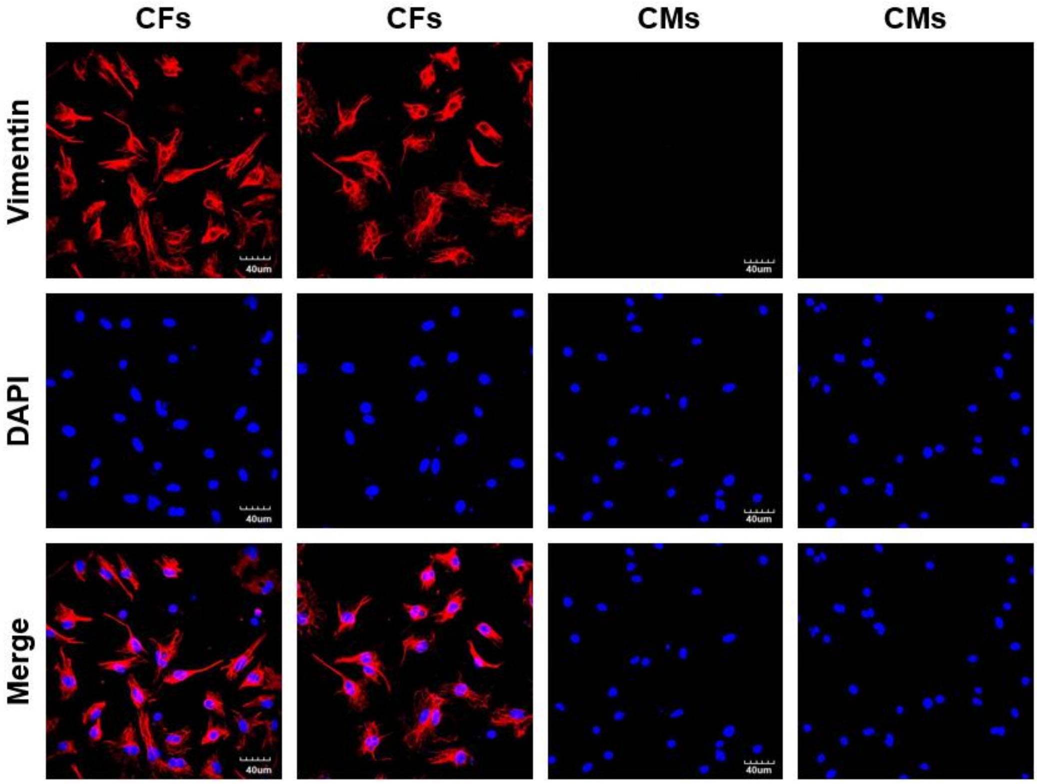

Fig. S1 Immunofluorescence detection of Vimentin in cardiomyocytes (CM) and cardiac fibroblasts (CF). Red color indicates Vimentin, blue color (DAPI) indicates nucleus.
